# Protective Effects of Astaxanthin against Oxidative Stress: Attenuation of TNF-α-Induced Oxidative Damage in SW480 Cells and Azoxymethane/Dextran Sulfate Sodium-Induced Colitis-Associated Cancer in C57BL/6 Mice

**DOI:** 10.3390/md22100469

**Published:** 2024-10-12

**Authors:** Haifeng Zhang, Min Wang, Yu Zhou, Shaojie Bao, Feng Wang, Chunmei Li

**Affiliations:** 1 College of Tourism and Cuisine, Yangzhou University, Yangzhou 225127, China; zhanghf@yzu.edu.cn (H.Z.); 18252731485@163.com (M.W.); 13142695810@163.com (F.W.); 2 Engineering Research Center for Huaiyang Cuisine of Jiangsu Province, Yangzhou 225127, China; 3 Key Laboratory of Chinese Cuisine Intangible Cultural Heritage Technology Inheritance, Department of Culinary Science, Ministry of Culture & Tourism, Yangzhou 225127, China; 4 College of Food Science and Engineering, Yangzhou University, Yangzhou 225127, China; zhouyuym1234@163.com (Y.Z.); mx120221247@stu.yzu.edu.cn (S.B.)

**Keywords:** astaxanthin, oxidative stress, inflammation, colitis-associated cancer

## Abstract

In this study, we investigated the protective effects of astaxanthin (AST) against oxidative stress induced by the combination of azoxymethane (AOM) and dextran sulfate sodium (DSS) in colitis-associated cancer (CAC) and TNF-α-induced human colorectal cancer cells (SW480), as well as the underlying mechanism. In vitro experiments revealed that astaxanthin reduced reactive oxygen species (ROS) generation and inhibited the expression of Phosphorylated JNK (P-JNK), Phosphorylated ERK (P-ERK), Phosphorylated p65 (P-p65), and the NF-κB downstream protein cyclooxygenase-2 (COX-2). In vivo experiments showed that astaxanthin ameliorated AOM/DSS-induced weight loss, shortened the colon length, and caused histomorphological changes. In addition, astaxanthin suppressed cellular inflammation by modulating the MAPK and NF-κB pathways and inhibiting the expression of the proinflammatory cytokines IL-6, IL-1β, and TNF-α. In conclusion, astaxanthin attenuates cellular inflammation and CAC through its antioxidant effects.

## 1. Introduction

Oxidative stress refers to the deleterious effects of highly reactive oxidizing molecules, either endogenously or exogenously generated, on living cells, which can be both free and non-free radicals [1]. They are able to readily acquire electrons from the molecules they come in contact with, thereby generating reactions that ultimately lead to the destruction of cellular structures [2]. Among these molecules, reactive oxygen species (ROS) and reactive nitrogen species (RNS) are produced endogenously at the highest concentrations and have major biological effects [3]. ROS are essential for biological processes. In normal cells, catalase (CAT), glutathione peroxidase (GSH-PX), and superoxide dismutase (SOD) partially help to clear ROS and maintain cellular redox homeostasis [4]. Excessive ROS production leads to oxidative stress [5]. Physiological levels of ROS are required for different processes, including intracellular signal transduction; metabolic, immune, and hypoxic responses; and transcriptional regulation [6]. At relatively high levels of ROS, the oxidation of proteins and DNA, among others, leads to cellular dysfunction [7]. Excess ROS may be pathological and lead to the development and progression of chronic diseases [8]. Oxidative stress and inflammation are densely separable [9]. Severe cases can lead to other related diseases such as intestinal inflammation [10,11] and colorectal cancer [12]. Therefore, there is an urgent need to prevent the harm caused by oxidative stress to prevent the development of colitis and colitis-related cancers.

Colorectal cancer (CRC) is the third most common cancer in the world and has become a global health concern [13]. Inflammatory bowel disease (IBD) is a chronic and recurrent inflammatory disease characterized by severe intestinal damage and an intense inflammatory response. The incidence of colorectal cancer in patients with IBD is almost three times greater than that in normal patients [14]. CAC is a major subtype of colorectal cancer closely associated with chronic or dysregulated inflammation that is difficult to treat [15]. CAC is a classic disease that transforms chronic inflammation into a tumor and results in long-term colitis in IBD patients [16]. Inside the epithelium, the mucus secreted by the cup cells acts as a barrier against the penetration of bacteria and pathogens. This gel coating facilitates the maintenance of a balanced intestinal environment. During colitis, the integrity of the coating is compromised, thereby allowing pathogens to contact the underlying immune system deep within the stroma [17]. Epidemiological studies have shown that anti-inflammatory treatments are effective in minimizing the incidence of CAC in patients with IBD [18]. Moreover, oxidative damage has been shown to be critical in the development of chronic inflammation [19]. Drugs that can inhibit cancer, such as 5-FU, oxaliplatin, and calcium folinate, can be used alone or in combination in the clinic. However, significant side effects of these drugs have been reported [20]. In recent years, extracts of natural substances have gained popularity as a class of cancer prevention drug. Astaxanthin (AST) has various functions, such as antioxidant, anti-inflammatory, and antidiabetic properties [21]. Many studies have shown that carotenoids, including AST, have potent cancer-preventive effects [22].

AST is a carotenoid extracted from shrimp, crabs, and seaweed, especially from *Haematococcus pluvialis* [23]. AST contains unsaturated hydroxyl groups, ketones, and conjugated double bonds, which have strong electronic effects and can bind to free radicals, thus scavenging free radicals and preventing oxidative damage [24]. AST has attracted scientific research attention due to its strong antioxidant properties [25]. The European Food Safety Authority recommends an acceptable daily intake of 0.034 mg/kg AST and concludes that the safety of daily intake of 0.06 mg/kg AST has not been fully determined [26]. However, several studies have shown that supplementing AST at doses higher than 0.06 mg/kg per day does not lead to any adverse reactions [27]. Owing to its unique molecular structure, AST can pass through the cell membrane barrier to reach its point of action and alleviate inflammation, cancer, obesity, diabetes, and other diseases through various biological applications [28]. In a previous study, AST significantly reduced the expression of key genes in the inflammation-related signaling pathway induced by oxidative DNA damage and suppressed inflammation [29]. In addition, AST inhibited dimethylhydrazine-induced colon carcinogenesis in rats by regulating the expression of nuclear factor-κB, COX-2, matrix metalloproteinase 2/9, proliferating cell nuclear antigen, and ERK [30]. Similarly, AST has also been shown to attenuate the severity of intestinal damage in patients with necrotizing small bowel colitis [31]. The effect of AST on patients with CAC has not been extensively studied. This study selected an appropriate concentration of AST for treatment on the basis of the results of preliminary experiments. The aim of this study was to investigate the therapeutic effects of AST on TNF-α-induced human colorectal cancer cells (SW480) and DSS/AOM-induced CAC in mice and to further explore its mechanism of action.

## 2. Results

### 2.1. Effects of AST on TNF-α-Induced SW480 Cells

#### 2.1.1. AST Attenuates TNF-α-Induced Oxidative Stress in SW480 Cells

**Figure 1:**
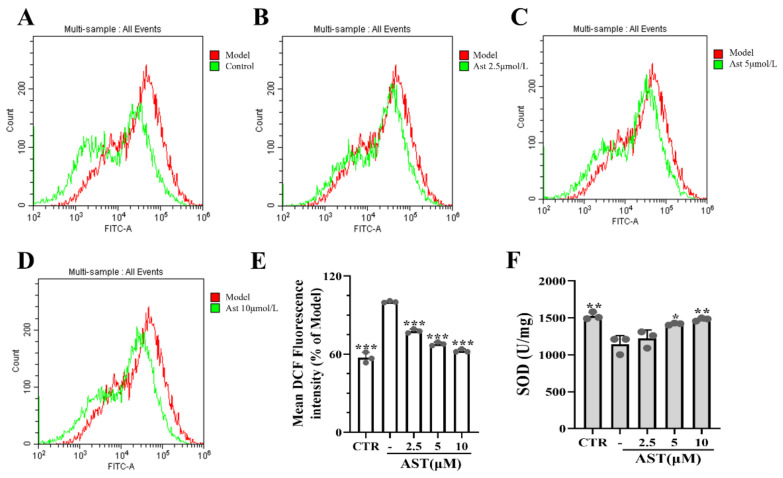
Effect of AST treatment on oxidative stress in SW480 cells induced by 50 μg/L TNF-α for 24 h. (**A**–**D**) DCF overlay histogram of each group and model group. (**E**) The fluorescence intensity of DCF. (**F**) SOD activity in different groups. CTR was the control group, - was the TNF-α model group, and 2.5–10 was the AST treatment group. After the cell modeling intervention was completed, cell proteins were extracted from the cell lysate for follow-up experiments. *p* values < 0.05 were regarded as statistically significant; * *p* < 0.05, ** *p* < 0.01, *** *p* < 0.001.

As shown in Figure 1, TNF-α-treated cells exhibited elevated ROS levels (*p* < 0.001, Figure 1A–E) and significantly decreased SOD activity (*p* < 0.01, Figure 1F). Interestingly, the ROS levels and SOD activity recovered in a concentration-dependent manner after AST treatment. These results indicated that AST can reduce oxidative stress.

#### 2.1.2. AST Inhibits TNF-α-Induced MAPK and NF-κB Signaling Pathways in SW480 Cells

**Figure 2:**
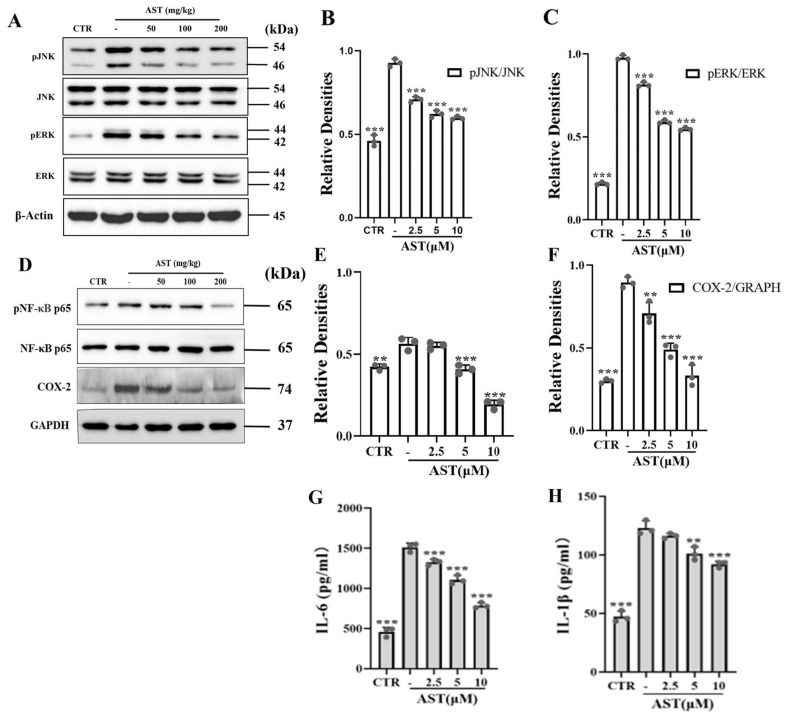
Effect of AST treatment on SW480 cells induced with 50 μg/L TNF-α for 24 h. (**A**) Western blot analysis of p-JNK, p-ERK, and β-actin. (**B**,**C**) Quantification of the protein expression of p-JNK, p-ERK, and β-actin was used as an internal control. (**D**) Western blot analysis of p-p65, COX-2, and GAPDH. (**E**,**F**) The quantification of the protein expression of p-p65 and COX-2; GAPDH was used as an internal control. (**G**,**H**) Contents of proinflammatory cytokines IL-6 and IL-1β. CTR was the control group, - was the TNF-α model group, and 2.5–10 was the AST treatment group. After the cell modeling intervention was completed, cell proteins were extracted from cell lysate for follow-up experiments. *p* values < 0.05 were regarded as statistically significant. ** *p* < 0.01, *** *p* < 0.001.

Compared with AOM/DSS alone, TNF-α increased the expression of P-JNK (*p* < 0.001) and P-ERK (*p* < 0.001), which decreased in a concentration-dependent manner after 24 h of AST treatment (Figure 2A–C). Similarly, TNF-α increased the expression of p-p65 (*p* < 0.01) and the NF-κB downstream protein COX-2 (*p* < 0.001), whereas AST treatment decreased their expression (Figure 2D–F).

#### 2.1.3. AST Downregulates TNF-α-Induced Inflammatory Cytokine Expression in SW480 Cells

To explore whether AST regulates the production of proinflammatory cytokines, the levels of IL-6 and IL-1β were measured. The results revealed that the levels of IL-6 (*p* < 0.001) and IL-1β (*p* < 0.01) were significantly increased in TNF-α-treated SW480 cells and gradually decreased with increasing concentrations of AST (Figure 2G,H).

### 2.2. Effects of AST on AOM/DSS-Induced Colon Cancer in Mice

#### 2.2.1. Effects of AST on Physical Changes, Colon Tissue Morphology, and Colon Length

**Figure 3:**
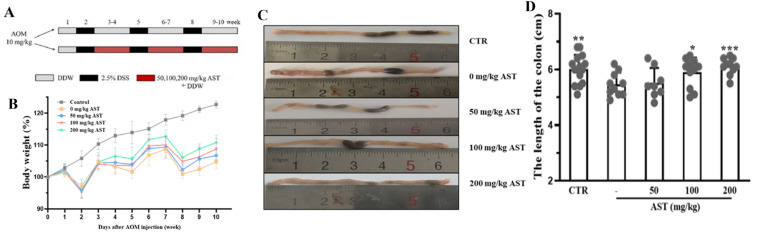
The CAC model-building method and the effect of AST in mice. (**A**) A schematic diagram of the model process and AST intakes. (**B**) Mouse weight map. (**C**) Colon tissue morphology. (**D**) Colon tissue length diagram. CTR was the control group, - was the AOM/DSS model group, and 50–200 mg/kg was the AST dose group. *p* values < 0.05 were regarded as statistically significant. * *p* < 0.05, ** *p* < 0.01, *** *p* < 0.001.

A CAC model was established in AOM/DSS mice, which were then treated with AST by gavage (Figure 3A). The colon length was lower in the AOM/DSS model group than in the control group (*p* < 0.001). The colon length gradually increased with increasing AST concentration (Figure 3B). After the mice were euthanized, the colonic tissue was collected. Compared with those in the control group, AOM/DSS-treated mice had severely more congested tissues with larger and denser tumors (Figure 3C); however, this effect was significantly attenuated by AST treatment. The improvement was even more pronounced in the high-dose AST group, with a trend toward a significant decrease in tumor number and density. The weights of the mice in all the groups decreased, and some of the mice had bloody stools, erect hair, poor mental status, and slow movement during the DSS cycle. After AST treatment, the weight recovered in a dose-dependent manner (Figure 3D).

#### 2.2.2. Hematoxylin-Eosin (HE) Staining of Mouse Intestinal Tissues

**Figure 4:**
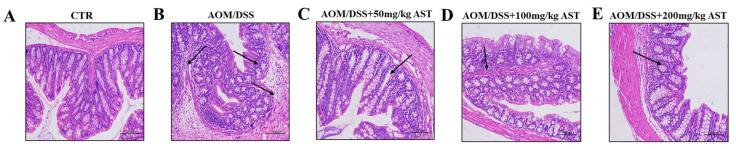
The CAC model-building method and the effect of AST in mice. (**A**–**E**) HE staining of colonic tissue. The arrow indicates the infiltration of inflammatory cells, enlarged voids, the disappearance of crypts, and the detachment of goblet cells. CTR was the control group.

HE staining was used to observe the morphological structure of the intestinal tissues. As shown in Figure 4, lymphocyte infiltration was detected in the mouse sections. The colonic glands of the normal group were neatly arranged and basically equal in shape and size. The epithelial mucosa and crypts were normal. However, in the AOM/DSS model group, the inflammatory features of the intestinal submucosa worsened, with connective tissue hyperplasia and severe lymphocytic infiltration. In addition, atrophy of the intestinal glands and the absence of crypts were also observed.

#### 2.2.3. AST Inhibits Ki67 Expression in AOM/DSS-Induced Mice

**Figure 5:**
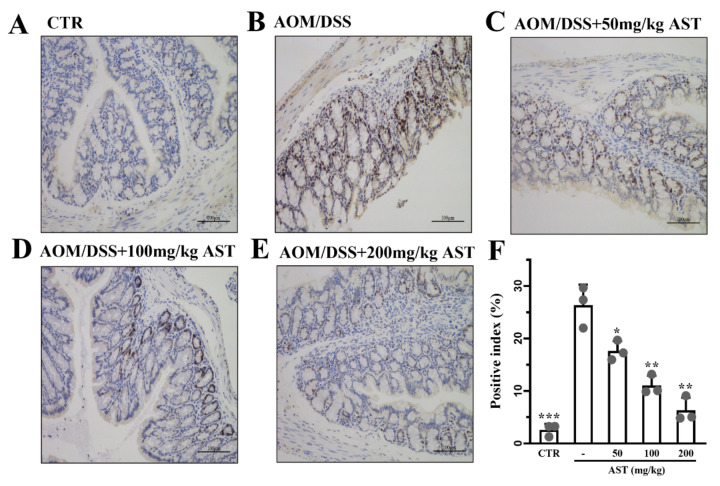
Effect of AST on Ki67 expression in mouse colorectum by immunohistochemistry analysis. (**A**–**E**) The immunohistochemistry graphs of Ki67 expression. (**F**) The positive index of Ki67. *p* values < 0.05 were regarded as statistically significant. Compared with the model group, CTR was the control group, - was the AOM/DSS model group, and 50–200 mg/kg was the AST dose group. * *p* < 0.05, ** *p* < 0.01, *** *p* < 0.001.

The expression of Ki67 was significantly increased in the AOM/DSS model group and decreased in the AST-treated group (*p* < 0.001, Figure 5A–F). In addition, the proportion of positive cells gradually decreased with increasing AST concentration. AST reduced the expression of Ki67 and inhibited the malignant proliferation of colon cancer cells.

#### 2.2.4. AST Attenuates the MAPK and NF-κB Pathways in AOM/DSS-Induced Mice

**Figure 6:**
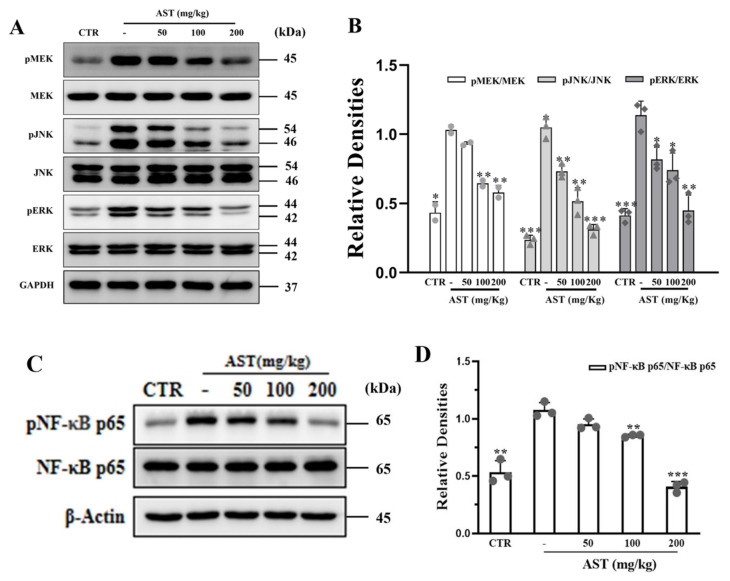
Effects of AST on MAPK and NF-κ B in mice. (**A**) Western blot analysis of p-MEK, p-JNK, p-ERK, and GAPDH. (**B**) The quantification of the protein expression of p-MEK, p-JNK, and p-ERK; GAPDH was used as an internal control. (**C**) Western blotting of p-p65 and β-actin. (**D**) The quantification of the protein expression of p-p65; β-actin was used as an internal control. CTR was the control group, - was the AOM/DSS model group without treatment, and 50–200 mg/kg was the AST dose group. *p* values < 0.05 were regarded as statistically significant. * *p* < 0.05, ** *p* < 0.01, *** *p* < 0.001.

To investigate the immunoregulatory mechanism of AST in AOM/DSS-induced CAC, we examined the expression of several key proteins of the MAPK and NF-κB pathways. The results showed that the expression of P-MEK (*p* < 0.05), P-JNK (*p* < 0.001), and P-ERK (*p* = 0.001) was significantly increased in the AOM/DSS group (Figure 6A,B). After AOM/DSS induction, the phosphorylation level of the inflammation-related protein NF-κB p65 in mouse colon tissue was upregulated, and the phosphorylation level of this protein gradually decreased with increasing AST concentration. (Figure 6C,D).

#### 2.2.5. AST Reduces Inflammatory Cytokines in AOM/DSS-Induced Mice

**Figure 7:**
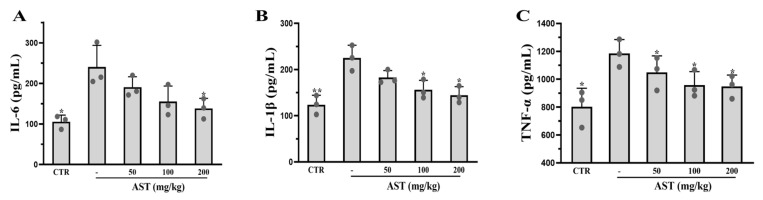
Effects of AST on downstream proinflammatory cytokines in mice. (**A**–**C**) The contents of proinflammatory cytokines IL-6, IL-1β, and TNF-α in mice. CTR was the control group, - was the AOM/DSS model group without treatment, and 50–200 mg/kg was the AST dose group. *p* values < 0.05 were regarded as statistically significant. * *p* < 0.05, ** *p* < 0.01.

The ELISA results revealed that the expression of IL-6 (*p* < 0.05), IL-1β (*p* < 0.01), and TNF-α (*p* < 0.05) was greater in the AOM/DSS group than in the control group. The expression of these proinflammatory cytokines was reduced in AST-treated mice (Figure 7A–C).

## 3. Discussion

Our main findings reveal the protective effect of astaxanthin on the excessive inflammatory response in SW480 cells and mice. Specifically, astaxanthin significantly reduced oxidative stress levels; significantly decreased the phosphorylation of JNK and ERK; and lowered the levels of IL-1β, IL-6, and TNF-α.

Our findings are consistent with previous research results. Astaxanthin has anti-inflammatory effects on a variety of diseases, such as Alzheimer’s disease, diabetes, acute kidney injury, cardiovascular disease, and pancreatic cancer [32,33,34,35]. The in vivo and in vitro results described in this study reveal the potential mechanism by which astaxanthin inhibits colitis.

The inflammatory response is a key component of the immune system, and unresolved inflammation can promote the occurrence of chronic diseases [36,37]. Confirmed inflammatory biomarkers include cytokines, transcription factors, and growth factors, as well as the NF-κB and MAPK pathways [38,39]. In addition to inflammatory biomarkers, the expression of NF-κB-related pathways and downstream inflammatory factors, including IL-1β, IL-6, and TNF α, are also affected by astaxanthin [40,41]. In addition, astaxanthin has been shown to affect MAPK signal transduction by regulating the expression of extracellular signal-regulated kinases and terminal kinases [42,43].

Colon length is often used as a marker of the degree of inflammation in individuals with colitis [44]. The results of HE staining indicated that AST intervention attenuated these inflammatory phenomena. In addition, the immunohistochemical parameter Ki67 is a nuclear-expressed protein consisting of two polypeptide chains coupled to a semiconserved replication of cellular DNA. Ki67 is considered an objective indicator of the overall proliferative activity of a cellular population [45]. Ki67 is hardly expressed in nonproliferative or low-transforming tissues but is expressed only in actively proliferating cells. The expression of Ki67 can be used as a biomarker to determine the malignancy and prognosis of *E. coli* tissue-associated cancers. In this study, we found that AST could reduce the expression of Ki67 and thus inhibit the proliferation of cancer cells, and in vivo experiments further verified that AST could inhibit the production of inflammatory factors and reduce the incidence of CAC by inhibiting the expression of proteins related to the MAPK and NF-κB pathways.

The MAPK signaling pathway is crucial for cell proliferation, differentiation, and apoptosis [46]. κB also plays a crucial role in regulating the immune response to infection. These results suggest that AST attenuates oxidative stress and reduces inflammation by inhibiting the expression of proteins related to the MAPK and NF-κB pathways as well as the production of proinflammatory factors. These findings are consistent with many previous findings that astaxanthin can inhibit the development of precancerous lesions in colon cancer by reducing oxidative stress, alleviating chronic inflammation, and inhibiting NF-κB activation and colon mucosal cell proliferation [47]. Astaxanthin can inhibit cell proliferation, migration, and invasion and induce cell cycle arrest and apoptosis by affecting MAPK, NF-κB, MMP, and apoptosis factors [48]. Research has also confirmed that astaxanthin regulates autophagy by modulating AMP-activated protein kinase, which is a cellular homolog of the mouse thymoma virus Akt 8 oncogene and MAPK [49]. Previous studies have shown that astaxanthin significantly reduces the expression of proinflammatory cytokines and enhances cell apoptosis induced by lipopolysaccharides in human neutrophils, thereby protecting against neutrophil inflammation [50]. In addition, astaxanthin can improve the gut microbiota and effectively treat DSS-induced acute colitis and chronic colitis-related intestinal fibrosis [51]. AST can also reduce free radicals and protect cells from oxidative damage in the synergistic treatment of inflammation [52].

Consistent with previous findings, treatment with S-MVLS significantly downregulated the expression of proinflammatory factors and significantly increased the expression of anti-inflammatory factors in the colonic mucosa, thereby improving DSS-induced colitis in mice [53]. Astaxanthin can protect against neuronal damage caused by Alzheimer’s disease by targeting the miR-7/SNCA axis to inhibit endoplasmic reticulum stress [54].

## 4. Materials and Methods

### 4.1. Reagents and Materials

High-glucose DMEM was purchased from Gibco (Grand Island, NY, USA); fetal bovine serum was obtained from EallBio (Beijing, China); penicillin-streptomycin was purchased from Beyotime Biotechnology (Beijing, China); AOM (A5486) and astaxanthin were provided by Sigma; DSS was obtained from MP (Santa Ana, CA, USA); consumables, SDS and HE staining reagents, were provided by Solarbio (Beijing, China); NCM Biotech (Suzhou, China) provided the protease and phosphatase inhibitor mixture, an antibody dilution solution, a rapid sealing solution, and protein blotting stripping solution, as well as an ultrasensitive ECL chemiluminescence kit and a high-sensitivity ECL chemiluminescence kit; the mouse IL-6, IL-1β, and TNF-α ELISA kits were provided by Mlbio (Shanghai, China). Protein markers and a rapid gel preparation kit were purchased from Vazyme (Nanjing, China). The primary antibodies (1:1000) included total MEK 1/2 (#9126), p-MEK 1/2 (#3958), total JNK (#9252), p-JNK (#4668), total ERK1/2 (#4695), p-ERK 1/2 (#4370), GAPDH (#5174), β-Actin (#4970), and all antibodies above were acquired from Cell Signaling Technology(Danvers, MS, USA), and NF-κB p65 and p-NF-κB p65 antibodies were sourced from Upstate Biotechnology (Shanghai, China). The secondary antibodies (1:5000) included Anti-rabbit IgG, HRP-linked Antibody (7074S) and Anti-mouse IgG, HRP-linked Antibody (7076S) sourced from Cell Signaling Technology.

### 4.2. In Vitro Experiments

#### 4.2.1. Cell Culture and Treatment

SW480 cells were cultured in complete DMEM (Gibco, Paisley, UK) supplemented with 10% fetal bovine serum (FBS) (EallBio, Beijing, China) and 1% penicillin-streptomycin (Beyotime, Shanghai, China) at 37 °C in a 5% CO_2_ cell culture chamber. SW480 cells were inoculated into a 96-well plate at a density of 1 × 10^4^. A control group, a model group, and a treatment group were established. The control group was treated with conventional culture medium, while the model group was treated with 50 μg/L TNF-α for 24 h. After pretreatment with 50 μg/L TNF-α for 2 h, each treatment group was added with different concentrations (2.5, 5, 10 μM) of AST for culture. When both model and drug cells were exposed to TNF-a, AST was used to intervene with the drug group. Finally, cell lysate was used to extract cell protein for subsequent experiments.

#### 4.2.2. Oxidative Stress Analysis

SOD activity was measured with a superoxide dismutase assay kit (Beyotime, Shanghai, China). The protein concentration was measured with a BCA assay kit (Thermo Fisher Scientific, Waltham, MA, USA). Briefly, the reagent solutions were added according to the instructions, followed by incubation at 37 °C for 30 min and detection of the absorbance at 450 nm. ROS levels were detected with an ROS assay kit (Beyotime, Shanghai, China). Briefly, 10 μmol/L DCFH-DA was added and the mixture was incubated at 37 °C for 20 min. Then, the cells were washed three times with PBS (centrifuged at 3000× *g* for 5 min at 4 °C) and analyzed via flow cytometry (Beckman, Germany).

### 4.3. In Vivo Experiments

#### 4.3.1. Establishment of the AOM/DSS-Induced Mouse Colon Cancer Model

Healthy C57BL/6 mice (weighing 18–20 g, 6–8 weeks old) were kept in the SPF-grade mouse house of the Centre for Comparative Medicine of Yangzhou University, License No. SYXK(SU)2017-0044. Mice were acclimatized for one week, and the test was started in the second week. Good ventilation and a clean and hygienic rearing environment were maintained in the mouse house, and the ambient temperature was maintained at 21~26 °C, the relative humidity was maintained at 40~70%, and the light exposure cycle was 12 h. All tests were completed in the light phase. Seventy-five mice were randomly divided into five groups, the blank control group, AOM/DSS model group, AST low-dose group (50 mg/kg), AST medium-dose group (100 mg/kg), and AST high-dose group (200 mg/kg), and the clipped toes of each mouse were numbered from 1 to 75. The blank control group was kept normally without other treatments; the AOM/DSS model group was treated with AOM and DSS; and the AST dose group was treated with AOM and DSS and gavaged with different concentrations of AST.

AOM (25 mg) was prepared with 2.5 mL of saline to make a 10 mg/mL solution, which was stored in a refrigerator at −20 °C. Before use, the solution was diluted with saline to a concentration of 1 mg/mL and mixed with sufficient shaking. On the first day of the experiment, all the mice were weighed with an electronic balance, and the mice in the model group and the AST dose groups were injected intraperitoneally with the corresponding volume of AOM at a final concentration of 10 mg/kg. The blank control group was injected intraperitoneally with physiological saline as a control. DSS was prepared as a 2.5% solution in the drinking water of experimental animals and dissolved by sufficient shaking. AST was dissolved in olive oil at the corresponding concentration, and the model group was simultaneously gavaged with olive oil as a control. Throughout the modeling process, the body weights of the mice were measured once a week, and the mice were sacrificed after the 10th week.

#### 4.3.2. Blood and Tissue Collection from Mice

The mice were anesthetized and killed by removing the eyeballs and collecting blood in centrifuge tubes. The serum was separated after the blood had been left to stand for 1 h, and then refrigerated at −80 °C. The colonic tissues of the mice in each group were fixed in 4% paraformaldehyde (Xianyang, China) at room temperature for 24 h. The collected tissue samples were then embedded in paraffin wax, serially sliced into 4 μm thick sections, and stained with hematoxylin and eosin (H&E) for morphological analysis [55]. The tissues were also collected and processed for further protein blot analysis.

#### 4.3.3. Inflammatory Factor Assay

Mouse plasma was centrifuged at 3000× *g* for 10 min, after which, the supernatant was collected. The levels of the inflammatory factors IL-6, IL-1β, and TNF-α in mouse serum were detected via a mouse ELISA kit [56]. The absorbance was measured at 450 nm using a microplate reader (BioTek, Burlington, VT, USA). A standard curve for the inflammatory factor assay was plotted, and the serum IL-6, IL-β, and tumor necrosis factor-α (pg/mL) levels in the mice were calculated.

### 4.4. Protein Blot Analysis

Proteins were extracted from intestinal tissues or cell lysates according to the manufacturer’s instructions. Proteins were separated on 10% separating gel, and at the end of electrophoresis, the proteins were transferred to a membrane transfer solution for transfer to a PVDF filter membrane. The membrane was blocked in skim milk for one hour, and after being washed with TBST, the membrane was incubated with a specific primary antibody (1:1000) at 4 °C overnight. Afterwards, the PVDF membrane was removed from the primary antibody, washed with TBST buffer, and incubated on a shaker with the corresponding secondary antibody for 60 min [55]. Finally, the membrane was visualized with a chemiluminescence imaging system.

### 4.5. Data Statistics and Analysis

All the data are expressed as the mean ± standard deviation (mean ± sem). The differences among the groups were analyzed via a randomized block design analysis of variance with the Statistical Package for Social Sciences (SPSS) software and GraphPad 8 software for graphing. Differences in the model group compared with all other groups were analyzed by *t*-test. *p* < 0.05 (*), *p* < 0.01 (**), and *p* < 0.001 (***) (*n* = 3).

## 5. Conclusions

In summary, in vitro experiments revealed that AST can increase the activities of superoxide dismutase and catalase, which are able to reduce TNF-α-induced oxidative stress damage in SW480 cells. AST inhibited the expression of proteins related to the MAPK and NF-κB signaling pathways as well as the levels of proinflammatory factors to reduce the inflammatory response. In in vivo experiments, AST reduced the phosphorylation of MAPK signaling pathway-related proteins in intestinal tissues, which could attenuate the apparent pathological symptoms of intestinal inflammation and colonic injury in mice. In addition, AST downregulated the expression of Ki67, reduced the malignant proliferation of colon cancer cells, and inhibited the expression of the proinflammatory factors IL-6, IL-1β, and tumor necrosis factor-α, thereby attenuating the colitis-associated colon cancer injury induced by AOM/DSS in mice. AST attenuated oxidative stress injury, providing a new opportunity for therapeutic agents.

## Data Availability

Data will be made available on request.
